# A Self-Powered Lactate Sensor Based on the Piezoelectric Effect for Assessing Tumor Development

**DOI:** 10.3390/s24072161

**Published:** 2024-03-28

**Authors:** Jiayan Lin, Pengcheng Yuan, Rui Lin, Xinyu Xue, Meihua Chen, Lili Xing

**Affiliations:** 1 School of Physics, University of Electronic Science and Technology of China, Chengdu 611731, China; ljyjiayanlin@163.com (J.L.); 2021120101007@std.uestc.edu.cn (P.Y.); linrui1105@163.com (R.L.); xuexinyu@uestc.edu.cn (X.X.); 2 Department of Radiation Oncology, Radiation Oncology Key Laboratory of Sichuan Province, Sichuan Clinical Research Center for Cancer, Sichuan Cancer Hospital & Institute, Sichuan Cancer Center, Affiliated Cancer Hospital of University of Electronic Science and Technology of China, Chengdu 610041, China; chenmeihua@scszlyy.org.cn

**Keywords:** self-powered, piezoelectric effect, lactate sensing, enzymatic reaction, tumor analysis

## Abstract

The build-up of lactate in solid tumors stands as a crucial and early occurrence in malignancy development, and the concentration of lactate in the tumor microenvironment may be a more sensitive indicator for analyzing primary tumors. In this study, we designed a self-powered lactate sensor for the rapid analysis of tumor samples, utilizing the coupling between the piezoelectric effect and enzymatic reaction. This lactate sensor is fabricated using a ZnO nanowire array modified with lactate oxidase (LOx). The sensing process does not require an external power source or batteries. The device can directly output electric signals containing lactate concentration information when subjected to external forces. The lactate concentration detection upper limit of the sensor is at least 27 mM, with a limit of detection (LOD) of approximately 1.3 mM and a response time of around 10 s. This study innovatively applied self-powered technology to the in situ detection of the tumor microenvironment and used the results to estimate the growth period of the primary tumor. The availability of this application has been confirmed through biological experiments. Furthermore, the sensor data generated by the device offer valuable insights for evaluating the likelihood of remote tumor metastasis. This study may expand the research scope of self-powered technology in the field of medical diagnosis and offer a novel perspective on cancer diagnosis.

## 1. Introduction

Cancer, an intricate and multifaceted health concern, remains a substantial global challenge. Given the escalating incidence of cancer in today’s world, timely diagnosis and thorough analysis of malignancies are crucial for ensuring effective treatment and accurate prognostic assessment. Currently, prevalent methods for tumor diagnosis encompass marker-based, pathological, imaging, and endoscopic diagnostics [1,2,3,4]. Additionally, innovative technologies in medical diagnostics, such as optoelectronics, fluorophores, photoacoustic imaging, computed tomography, and magnetic resonance imaging [5,6,7,8,9], have been proposed by certain research groups and have the potential to be applied to cancer diagnosis. In recent years, significant strides have been made in the rapid advancement of nanotechnology and quantum-based nanomaterials, particularly in the fields of imaging and therapy [10,11,12,13,14], highlighting their potential applications in cancer diagnosis and analysis. Within the domain of nanomaterials, biosensors hinged on self-powered nanogenerators exhibit a diverse spectrum of application scenarios [15,16,17]. Consequently, the development of a novel self-powered auxiliary device capable of assessing tumors becomes necessary, facilitating the prompt analysis of biomarker components within the tumor microenvironment.

Circulating lactate is a key metabolic fuel source in both normal tissues and tumors [18]. Additionally, as a crucial metabolic product associated with tumor development, lactate has become a pivotal link between cancer cells and their microenvironment. The investigations of the specific mechanisms of lactate in tumor development have indicated the intricate role of lactate in cancer progression [19,20]. Among these, the build-up of lactate in solid tumors stands as a crucial and early occurrence in malignancy development. Furthermore, the measurement of lactate in primary tumors can serve as a foundation for new metabolic classifications and may be a more sensitive indicator for evaluating the invasiveness and metastatic potential of primary tumors, thereby improving the prognosis and treatment of clinical oncology [19,21,22]. Changes in lactate concentrations in the body can arise from human exercise as well as various diseases. Due to these complexities, low-invasive lactate sensors designed for sweat, urine, and tears may encounter challenges in accurately determining the stage of tumor development. In contrast, the in situ detection of the tumor microenvironment offers a higher degree of accuracy, rendering it a preferable option in cancer analysis. Currently, numerous lactate sensors have been proposed for clinical and biological applications, encompassing various technologies such as electrochemical [23,24,25], optical [26,27], and enzyme-catalyzed [28,29] methods. Among these, lactate biosensors based on the enzyme-catalyzed method stand out for their numerous advantages, including high selectivity, rapid measurement, mild reaction, and short sample preparation time [30], surpassing other sensors and demonstrating remarkable proficiency in swift measurements within intricate environments. Both lactate oxidase (LOx) and lactate dehydrogenase (LDH) exhibit enzymatic reactivity towards lactate. Notably, LOx possesses high specificity and sensitivity [31], and it has no need for supplementary co-enzymes [32], making it a more suitable candidate for biosensor applications.

In this article, the piezoelectric–enzyme coupling is innovatively introduced for the detection of lactate concentrations in the tumor microenvironment. We designed a self-powered lactate biosensor from ZnO nanowire arrays, for which its advantage is reflected in its fast detection, self-powered feature, and high specificity. The technology utilizes the piezoelectric effect of piezoelectric nanogenerators in conjunction with enzymatic reactions. It exploits the influence of LOx catalytic products on the piezoelectric properties of ZnO nanowires, translating lactate concentrations into an output voltage, thereby offering a novel approach for the efficient detection of lactate. Compared with other technologies present in the market for cancer diagnostics, the lactate sensor in this study adopts self-powered technology and an in situ detection strategy, which has the advantages of high accuracy and real-time cancer diagnosis. Self-powered technology allows the lactate sensor to function without external power or batteries [33,34]. The in situ extraction of lactate information from tumor tissues ensures a strong correlation between lactate concentration and the stage of cancer development, while effectively mitigating any confounding influences arising from other lactate-related physiological phenomena. The operation of in situ lactate extraction is very convenient, requiring only simple tissue segmentation, grinding, and centrifugation. After undergoing these processing steps, the resulting liquid can be subjected to ex vivo analysis using the lactate sensor, thereby enabling the detection of lactate concentration within the processed sample. Furthermore, due to the high selectivity and efficiency of enzymatic reactions, the biosensor can exclude the influence of other components in complex tumor microenvironments, allowing the rapid and specific monitoring of lactate concentration changes. The concentration of lactate in the tumor microenvironment detected by the sensor can be used to assess the stage of the primary tumor and provide a reference for evaluating the likelihood of tumor metastasis in the remote region. This achievement can assist doctors in the diagnosis of patients with cancer and further provide a reference for the choice of cancer treatment. This work can expand the research scope of self-powered technology in the field of medical diagnosis.

## 2. Experimental Section

### 2.1. Materials

Zinc acetate dihydrate (Zn(CH_3_COO)_2_·2H_2_O, CAS: 5970-45-6), ammonia solution (NH_3_·H_2_O, CAS: 1336-21-6), L-(+)-lactic acid (C_3_H_6_O_3_, CAS: 79-33-4), sodium hydroxide solution (NaOH, 1.0 M, CAS: 1310-73-2), D-(+)-glucose (C_6_H_12_O_6_, CAS: 50-99-7), uric acid (C_5_H_4_N_4_O_3_, CAS: 69-93-2), urea (CH_4_N_2_O, CAS: 57-13-6), sodium chloride (NaCl, CAS: 7647-14-5), lactate oxidase (LOx, 20 U/mg, CAS: 9028-72-2), and phosphate-buffered saline (PBS, 0.1 M, pH 7.2~7.4, CAS: NONE8646) were purchased from Shanghai Macklin Biochemical Technology Co., Ltd. (Shanghai, China).

### 2.2. Synthesis of ZnO Nanowire Arrays

ZnO nanowire arrays were hydrothermally synthesized and vertically aligned on a Ti substrate [35,36]. Initially, 38 mL of deionized water was used to dissolve 0.416 g of Zn(CH_3_COO)_2_·2H_2_O, which was then stirred for 10 min at room temperature. Subsequently, 2 mL of NH_3_·H_2_O was introduced into the solution. The Ti substrate was immersed in the solution, and the reaction mixture was maintained at 93 °C for 24 h with a sealed reaction breaker. Afterwards, the substrate underwent washing with deionized water and absolute ethanol, followed by drying at 60 °C. The resulting nanowire arrays exhibited vertical alignment on the Ti substrate.

### 2.3. Fabrication of Self-Powered Lactate Sensor

The Ti sheet, adorned with ZnO nanowires, was meticulously trimmed to a standardized dimension of 1.5 × 0.8 mm^2^. Subsequently, Cu foil was attached to the top of the nanowires and the back of the Ti sheet to form the electrode. Following this attachment, the device underwent encapsulation with a PI film, leaving a section of the ZnO nanowires exposed, enabling contact with the liquid to be tested. The exposed ZnO nanowires were modified with lactate oxidase (LOx). For the preparation of the LOx stock solution, 5.0 mg of LOx lyophilized powder was dissolved in 0.5 mL of PBS. To facilitate complete solubilization, manual agitation was employed, complemented by the repeated aspiration and ejection of the solution using a pipette [37]. The nanowires of the piezoelectric biosensor unit were subjected to a gradual dripping of the LOx solution. Subsequently, the incubation process took place within a ventilation cabinet, lasting for 2 h. Following two repetitions of this process, deionized water was employed to cleanse the device, eliminating excess LOx. After carrying out the preceding operational procedures, the lactate sensor was fully fabricated.

### 2.4. Characterization and Measurement

Scanning Electron Microscopy (SEM, Zeiss Gemini 300, Carl Zeiss, Jena, Germany) was utilized to examine the morphology and microstructure of ZnO nanowires. X-ray Diffraction (XRD, Philips X’ Pert Pro MPD, Almelo, the Netherlands) was employed to characterize the crystal phase of the ZnO nanowires.

The output piezoelectric voltage was monitored by a programmable electrometer (Model 6514, Keithley Instruments, Cleveland, OH, USA) and a standard commercial force sensor measured the force. To prepare the lactate solution, L-(+)-lactic acid was diluted to different concentrations and the pH was adjusted to 7.0 with the sodium hydroxide solution. The bio-marker concentration in the solution was precisely controlled through a rigorous preparation process. During the stability experiment, the device operated continuously for 600 cycles under the pressure of a fixed frequency and magnitude in the 9 mM lactate solution. In addition, the device remained immersed in the 9 mM lactate solution, with solution measurements taken at specific intervals (0, 26, 48, 71, and 96 h) under an applied force of 15 N. As for the biological experiments, we used the same method as before.

### 2.5. Cell Culture

The mouse breast cancer cell line (4T1) and mouse fibroblast cell line (L-929) underwent culture in RPMI 1640 medium (Yeasen, Shanghai, China) supplemented with 10% (*v*/*v*) FBS (Yeasen, Shanghai, China). 4T1-luc cells, expressing Luciferase, were generated by infecting 4T1 cells with lentiviruses encoding Luciferase (pLenti PGK V5-LUC Neo, Addgene plasmid #21471). Seeding 1 × 10^4^ cells per well in a 24-well plate (Corning Inc., New York, NY, USA), both 4T1 cells and L-929 cells were incubated at 37 °C/5% CO_2_ for 48 h. During 24 to 48 h of culture, the 4T1-luc supernatant was taken eight times at the same time interval. From 24 to 48 h of culture, the L-929 cell supernatant was taken twice at the same time interval.

### 2.6. Tumor Tissue Culture

As described previously [38], female BALB/c mice, aged 6–8 weeks and weighing 13–20 g, were subjected to a reverse light/dark cycle of 12/12 h. Food and water were available ad libitum. Anesthesia for surgery was induced using 1% sodium pentobarbital for the experimental mice. Post-surgery, the mice received an injection of an anti-inflammatory drug. In the right upper abdomen of each mouse, 0.1 mL of 4T1-luc tumor cells (1 × 10^6^ cells/mL) was inoculated. The mice were categorized into four groups, and daily monitoring commenced to observe tumor growth. When the tumor volume of the mice in the 4 groups was 100, 150, 300, and 800 mm^3^, respectively, the tumor was stripped and sampled for lactate detection.

All the mice received standard laboratory diets and water. The animals were housed under standard laboratory conditions (21 °C ± 2 °C, 12 h of darkness/12 h of light) [39]. Animal experiments were conducted in accordance with the Principles of Laboratory Animal Care (the People’s Republic of China), and all animal experiments were performed in accordance with the Guidelines for the Care and Use of Laboratory Animals of the Ethics Committee for Medical Research and New Medical Technology of Sichuan Cancer Hospital (SCCHEC-04–2023–026).

## 3. Results and Discussion

**Figure 1:**
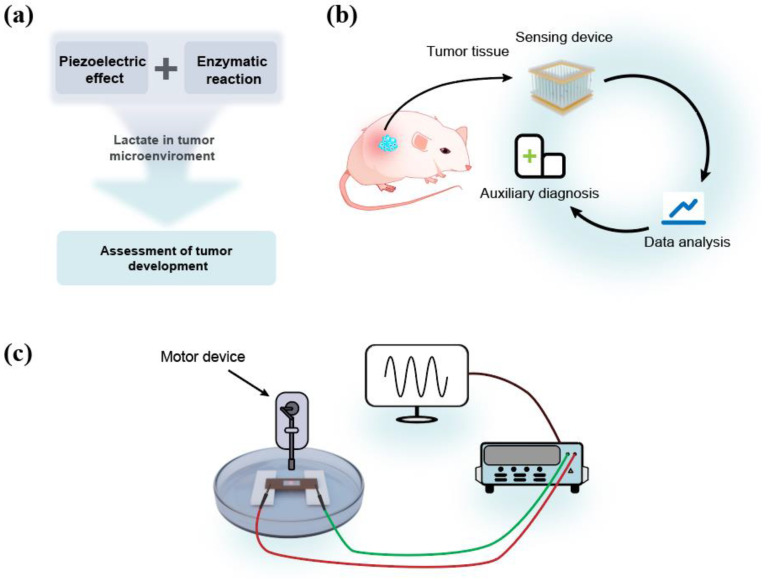
(**a**) The technical route of the sensor. (**b**) The potential application area of the sensor. (**c**) The measurement system.

Figure 1a shows the technical route of the self-powered lactate sensor for the assessment of tumor development. ZnO, as a third-generation semiconductor, exhibits both semiconductor and piezoelectric properties due to its lack of a symmetric center. Applying pressure along the c-axis of a ZnO nanowire results in the generation of piezoelectric polarization charges at its two ends, enabling the output of piezoelectric potential/current. Enzyme modification on the surface of ZnO leads to specific enzymatic reactions which affect the piezoelectric polarization charges of ZnO, thus influencing its piezoelectric outputs. Modifying lactate oxidase on the surface of ZnO enables the coupling of the piezoelectric effect and enzymatic reaction for actively detecting lactate content in tumor microenvironments, thereby facilitating the assessment of tumor development. Figure 1b shows the potential application of the self-powered lactate sensor. This biosensor is designed for auxiliary diagnosis in future medical processes. The detection of lactate in the vicinity of tumor tissue can assist in the analysis of tumor development during the diagnostic process. Figure 1c shows the measurement system for lactate detection. After immersing in the test solution containing lactate, piezoelectric outputs of the sensor can be detected by an electrometer, which is driven by a programmable stepper motor [15,16]. Detailed experimental methods can be found in the Experimental Section.

**Figure 2:**
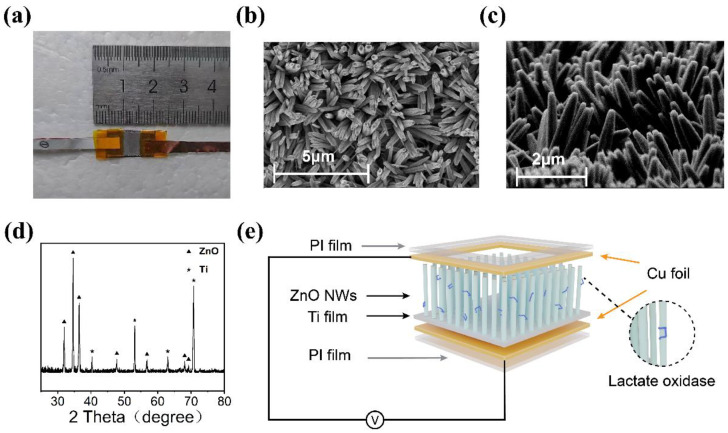
(**a**) Photograph of the device. (**b**) The top-view SEM image of ZnO nanowire array. (**c**) The side-view SEM image of ZnO nanowire array. (**d**) XRD pattern of the ZnO nanowire array on Ti substrate. (**e**) The structure of the device.

Figure 2 displays the characterization of ZnO nanowire arrays and the device. Figure 2a shows the photograph of the device, characterized by compact dimensions designed to suit its intended functionality. The size of the core sensing part of the device is about 0.5 × 0.8 mm^2^, and the overall size of the device is about 2.5 × 0.8 mm^2^. Figure 2b presents the top-view SEM image of the nanoarray, depicting nanowires with a hexagonal structure and an average diameter of ~200 nm. Figure 2c illustrates the side-view SEM image, showcasing vertically aligned nanowires with an average length of 2 μm grown on the substrate. Figure 2d presents the XRD pattern of the ZnO nanowires. The diffraction peaks corresponding to the triangular pattern represent ZnO (JCPDS #36-1451), while the peaks associated with the pentagram pattern represent Ti (JCPDS #44-1294). Among them, the seven peaks at 31.936°, 34.602°, 36.400°, 47.732°, 56.779°, 68.044°, and 69.226° correspond to the (100), (002), (101), (102), (110), (112), and (201) crystal planes of ZnO, respectively. The presence of sharp diffraction peaks suggests good crystalline quality. ZnO nanowires and the Ti substrate as the base have proven to have good biocompatibility [40,41,42]. Figure 2e displays the structure of the sensor. The sensor employs Cu foil as upper and lower electrodes and is encapsulated using a PI film. Due to the gaps between ZnO nanowire arrays, solutions used for testing, as well as enzyme solutions used for enzyme modification, can directly contact the nanowires without microfluidic design.

**Figure 3:**
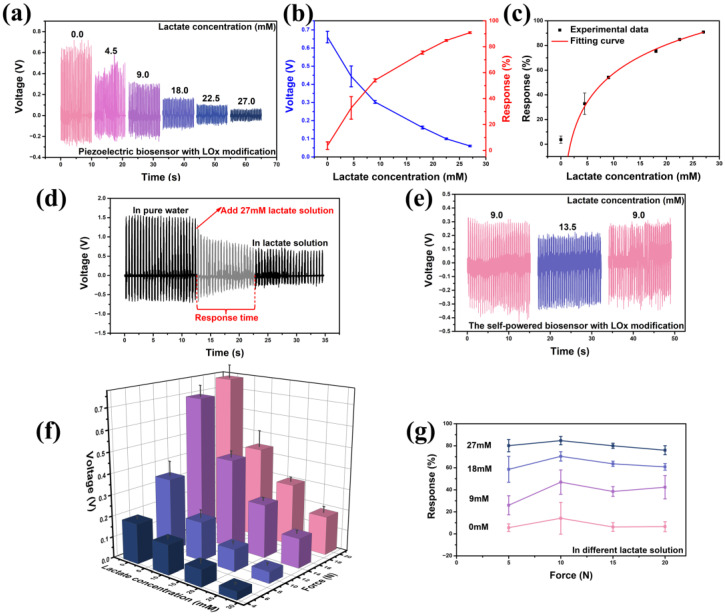
The lactate sensing capability of the self-powered sensor with LOx modification. (**a**) The output piezoelectric voltage under the force of 15 N and the frequency of 2 Hz in different lactate solutions. (**b**) The response in different lactate solutions. (**c**) The response’s fitting curve of the lactate sensor. (**d**) The change in the output piezoelectric voltage when the lactate solution with 27 mM was dropped into pure water. (**e**) The reversibility of the sensor. (**f**) The output piezoelectric voltage under different forces (5, 10, 15, and 20 N). (**g**) The sensor’s response to varying applied forces and lactate solutions with various concentrations.

The normal concentration of lactate in blood and healthy tissues typically ranges from 1.5 to 3 mM [43]. In contrast, cancer tissues may exhibit higher concentrations, ranging from 10 to 30 mM [44]. Therefore, the lactate concentration varied from 0 to 27 mM during the test. Figure 3 shows the sensing capability of the self-powered sensor designed for lactate detection. In Figure 3a, the piezoelectric voltage outputs of the LOx-modified sensor are presented under an applied force of 15 N and a frequency of 2 Hz in lactate solutions with different concentrations. Figure 3b showcases both the piezoelectric voltage output and the response of the self-powered lactate biosensor. The response of the sensor can be defined as follows [45]:

(1) $$R\%=\left|\frac{V_{0}-V_{i}}{V_{0}}\right|\times \;100\%\;$$

Here, $V_{0}$ and $V_{i}$ represent the piezoelectric voltages of the device in pure water and the test solution with varying concentrations of lactate, respectively. In 0, 4.5, 9, 18, 22.5, 27 mM lactate solutions, the output piezoelectric voltage is 0.661, 0.444, 0.303, 0.162, 0.100 and 0.060 V, and the response is 3.72, 32.80, 54.10, 75.47, 84.83 and 90.87%, respectively. By fitting the response scatter plot, the limit of detection can be evaluated to be ~1.3 mM [46]. In addition, as shown in Figure 3c, the response curve of the lactate sensor can be accurately fitted using the equation y=−19.84+33.54lnx, and the coefficient of determination (R-squared) is ~0.995, indicating excellent goodness of fit. The response time and change in the output piezoelectric voltage when the lactate solution was dropped into pure water are shown in Figure 3d. At the pressure of 30 N and a frequency of 2 Hz, the output piezoelectric voltage changed from 1.527 to 0.660 V after the 27 mM lactate solution was added, and it retained stability quickly within several seconds. The response time of the sensor is about 10 s, which is much slower than the rate of change in lactate concentrations in the tumor microenvironment. Table 1 presents a comparison between the present work and recently reported methods for lactate detection. It is evident from the comparison that the device designed in present work exhibits a significant advantage in response time. Furthermore, unlike other devices, the present design does not require an external power source, which is a major characteristic of the device.

Figure 3e shows that the device can detect both the increase and decrease in lactate concentration, demonstrating the good reversibility of the device. The sensor also retains stable sensing performance under different forces (Figure 3f,g). At the pressure of 5 N and a frequency of 0.5 Hz, the output piezoelectric voltage in 0, 9, 18, and 27 mM lactate solutions is 0.189, 0.140, 0.078, and 0.038 V. At the pressure of 10 N and a frequency of 0.5 Hz, the output piezoelectric voltage in 0, 9, 18, and 27 mM lactate solutions is 0.377, 0.216, 0.103, and 0.053 V. At the pressure of 15 N and a frequency of 0.5 Hz, the output piezoelectric voltage in 0, 9, 18, and 27 mM lactate solutions is 0.696, 0.428, 0.253, and 0.139 V. At the pressure of 20 N and a frequency of 0.5 Hz, the output piezoelectric voltage in 0, 9, 18, and 27 mM lactate solutions is 0.761, 0.439, 0.298, and 0.183 V. It is noteworthy that the response of the self-powered lactate sensor is nearly consistent under the different applied forces (Figure 3g), indicating its excellent flexibility and minimal restriction to applied forces. When the sensor is immersed in a 9 mM lactate solution, under the applied forces of 5, 10, 15, and 20 N, the response is 25.95, 46.98, 38.46, and 42.33%, respectively. When the sensor is immersed in an 18 mM lactate solution, under the applied forces of 5, 10, 15, and 20 N, the response is 58.59, 70.38, 63.62, and 60.84%, respectively. When the sensor is immersed in a 27 mM lactate solution, under the applied forces of 5, 10, 15, and 20 N, the response is 80.11, 84.68, 79.95, and 75.95%, respectively. In 9, 18, and 27 mM lactate solutions, the average response under different forces is 38.43, 63.35, and 80.17%, respectively. The estimation of lactate concentrations can be derived from the response curves.

**Figure 4:**
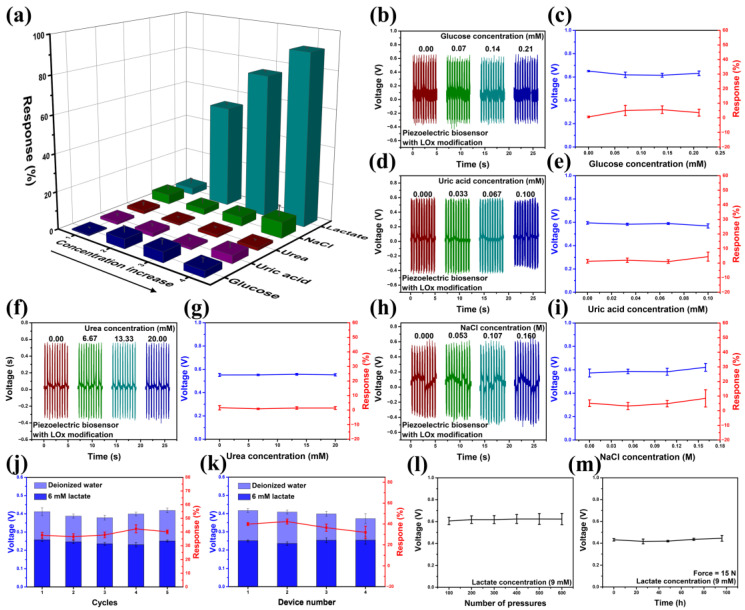
(**a**–**i**) The response of the device against glucose, uric acid, urea, and NaCl (force = 15 N). (**j**) Response–recovery cycles of a single lactate sensor (force = 10 N). (**k**) The output voltage and response of 4 lactate sensors manufactured using the method for illustrating reproducibility (force = 10 N). (**l**,**m**) The stability of the sensor in a 9 mM lactate solution.

The specificity of the device against the lactate solution is shown in Figure 4a–i. Glucose serves as a crucial metabolic substrate for all mammalian cells, with tumor cells exhibiting an augmented glucose metabolism compared to normal tissues [52]. Consequently, glucose concentration within the tumor microenvironment undergoes dynamic variations. Additionally, several studies have implicated the concentration of specific ions, such as Na^+^, in the tumor microenvironment as being associated with tumor progression [53]. Furthermore, traces of uric acid, urea, and other components are also present in the bodily environment. The specificity experiment demonstrates that the lactate sensor exhibits no significant response to these interfering components, and it can specifically indicate lactate concentration (Figure 4a). A device was utilized to repeatedly measure both deionized water and a 6 mM lactate solution. Over five cycles, the device exhibited a consistent output voltage and response (Figure 4j). The responses obtained from the five trials were 37.65, 36.57, 37.87, 42.32, and 40.17%, respectively, which demonstrated a close alignment with the fitting curve, thereby validating the high accuracy and reproducibility of a single lactate sensor. Figure 4k presents the output voltage and response of four lactate sensors, fabricated using the same method, in both deionized water and a 6 mM lactate solution. Notably, the performance of these four devices exhibits remarkable consistency. The manufacturing process of the sensors ensures rigorous control over the geometric dimensions of the sensor, the quantity of lactate oxidase, and other parameters, thereby ensuring outstanding consistency among the devices and reproducibility of multiple lactate sensor. Consequently, no additional calibration step is required. Figure 4l and m show the excellent stability of the sensor with LOx modification, which can continuously and stably output piezoelectric voltage in lactate solutions for more than 600 cycles under the pressure of a fixed frequency and magnitude provided by the stepper. And the sensor exhibits consistent piezoelectric output even after 100 h of continuous operation, demonstrating its excellent stability.

**Figure 5:**
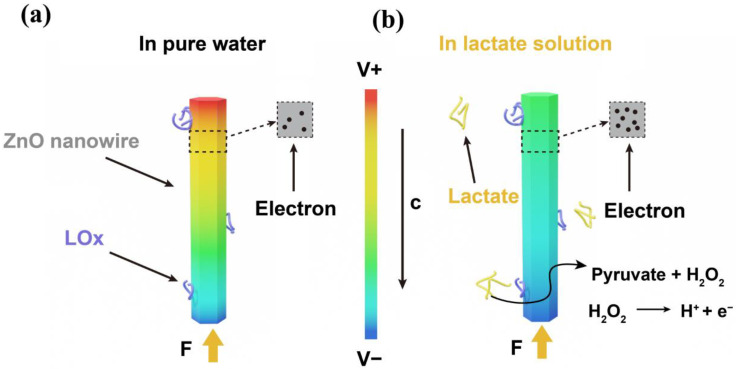
The sensing mechanism of the sensor. (**a**) The ZnO nanowire modified with LOx in pure water with an external force acting on the c-axis. (**b**) The ZnO nanowire modified with LOx nanowire in lactate water with an external force acting on the c-axis.

Figure 5 presents the sensing mechanism of the self-powered lactate sensor with LOx modifications. Figure 5a depicts the piezoelectric effect of the LOx-modified ZnO nanowire in the absence of the lactate solution. When a force is applied in the c-axis direction of the ZnO nanowire with LOx modification, the ZnO nanowire can be compressed and can output piezoelectric voltages [54]. According to research findings, the piezoelectric effect of ZnO is influenced by its surface carrier density [55]. In pure water, the ZnO nanowire does not come into contact with lactate, and thereby, no enzymatic reaction occurs. ZnO exhibits a low surface carrier density, resulting in high output piezoelectric voltages. In lactate solutions, a combined reaction of piezoelectric and enzymatic reactions takes place, with both processes occurring concurrently, as shown in Figure 5b. Here, LOx and lactate undergo enzymatic reactions (Equations (2) and (3)) [56].

(2) $$\mathrm{Lactate}+H_{2}O+\;O_{2}\overset{\mathrm{LOx}}{\to }\;\mathrm{pyruvate}+\;H_{2}O_{2},$$

(3) $$H_{2}O_{2}+O_{2}\overset{\mathrm{LOx}}{\to }{2H}^{+}+O_{2}\;+2e^{-}$$

$e^{-}$ and $H^{+}$ accumulate significantly as additional charge carriers on the surface of the ZnO nanowire [57]. This accumulation results in increasing the surface carrier density of ZnO, which can partly screen the piezoelectric effect of the ZnO nanowire. As more lactate molecules undergo enzymatic reactions catalyzed by lactate oxidase, this screen effect becomes more pronounced, resulting in a reduced piezoelectric output.

**Figure 6:**
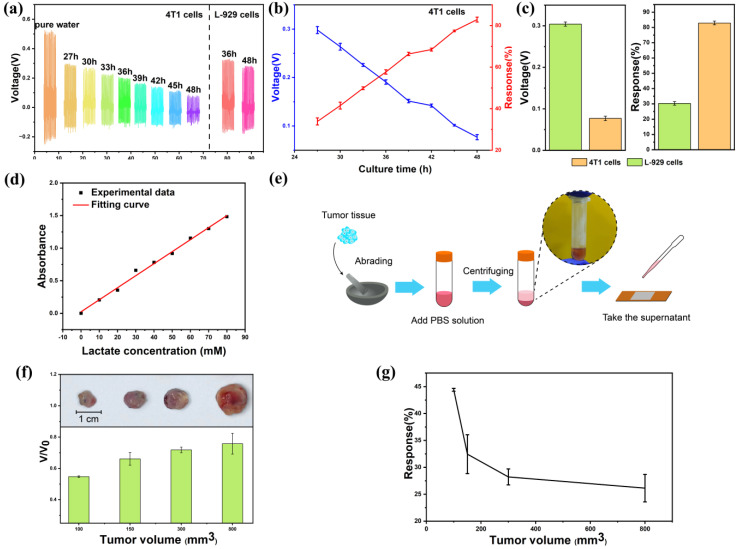
In vitro real-time human cancer cell lactate detection and in vitro mouse tumor sensing experiment. (**a**) The output piezoelectric voltage of the biosensor for the lactate sensing of cancer cells and normal cells of different culture time. (**b**) The response and output piezoelectric voltage of the biosensor for lactate sensing of cancer cells of different culture times. (**c**) The response and output piezoelectric voltage at 48 h of cancer cells and normal cells. (**d**) The experimental data and fitting curve for the lactate concentration measured by ultraviolet spectrophotometer. (**e**) Diagram of in vitro tumor experiments. (**f**) The photographs and normalized output piezoelectric voltage of the device for lactate sensing of tumors of different volumes (100, 150, 300, and 800 mm^3^). (**g**) The response of the device for lactate sensing of tumors.

Figure 6 shows the in vitro real-time cancer cell lactate detection and the in vitro mouse tumor sensing experiment. Based on the good performance of the self-powered biosensor in cell-free culture media, we further tested its practical medical application in detecting lactate secretion from cancer cells. Culture media from cancer cells (4T1-luc) cultured for 48 h and culture media from normal cells (L-929) were collected at certain intervals. Figure 6a shows the output piezoelectric voltage of the self-powered biosensor when detecting lactate in the collected culture media. In comparison to normal cells, the output voltage of the device demonstrates a stronger negative correlation with the incubation time of cancer cells. Figure 6b provides an overview of the average voltage and response variation in the device throughout the cancer cell incubation process. For cancer cells cultured for 27, 30, 33, 36, 39, 42, 45, and 48 h, the response is 33.85, 41.49, 49.84, 57.66, 66.38, 68.46, 77.46, and 82.92%, respectively. It can be observed that the response of the device increases gradually with the increase in cell-incubation time. This trend can be attributed to the accumulation of lactate during the culture of cancer cells. Figure 6c depicts the average voltage and response at 48 h generated by the device induced by cancer cells and normal cells. At the same culture time, the cancer cell media induced a higher response than the normal cell media, which should be due to the accelerated lactate efflux due to the cancer cells’ Warburg effect [58].

To further explore the preclinical application of the device, an in vitro tumor sensing experiment was conducted. The lactate concentration in the tumor changes with its volume. This sensor can determine the size of the tumor by detecting the lactate concentration, which is expected to further determine the stage of cancer. In this study, we selected tumors of different sizes (100, 150, 300, and 800 mm^3^) and measured their lactate concentrations. Figure 6d depicts the fitting curve for the lactate concentration determined using an ultraviolet spectrophotometer. The lactate concentration measured by the lactate sensor proposed in this study in a 4T1 cell culture medium (cultured for 48 h) was 21.41 mM, while the actual lactate concentration measured by an ultraviolet spectrophotometer was 20.99 mM, exhibiting a near-perfect alignment between the two methodologies. As shown in Figure 6e, we extracted lactate solutions from tumors of different sizes and used the self-powered biosensor for detection. Take 0.25 g tumor, grind it for 10 min and add 1 mL PBS solution. Centrifuge at 900 N for 8 min and add the supernatant droplets to the device for testing. In Figure 6f, the normalized piezoelectric voltage output of the device is presented across various tumor volumes. V represents the device’s piezoelectric voltage output corresponding to different tumor volumes, while V_0_ denotes the output in pure water. The output piezoelectric voltage of the device is negatively correlated with the tumor volume. Figure 6g shows the response variation in the device during the tumor volume growth process. For tumors with a volume of 100, 150, 300, and 800 mm^3^, the response is 44.41, 32.45, 28.22, and 26.14%, respectively, indicating a positive correlation between the tumor volume and the device response. The lactate accumulation in the early stages of cancer development was successfully observed by the self-powered piezoelectric biosensor in the tumor experiment.

Despite the commendable sensing performance demonstrated by the lactate sensor proposed in this study for tumor evaluation, there still exists ample room for further advancements. Although the sensor designed in this study does not require additional batteries itself, it still needs an external power source for recording the device’s output voltage. In the future, we will persist in investigating self-powered data acquisition, modulation, transmission, and storage, integrating them with the sensor proposed in this research to develop a comprehensively self-powered system throughout the entire process. Additionally, further optimizing the detection limit, sensitivity, and stability of this lactate sensor is crucial to accurately reflect the lactate concentration within the tumor microenvironment. During our experiments, tumor tissue was extracted and evaluated in vitro, yielding encouraging results. Given these favorable outcomes, one of our future research endeavors will be centered on the development of an implantable in vivo lactate sensor. Additionally, the biosafety and toxicity of the device also need to be further studied.

## 4. Conclusions

In this paper, we fabricated a novel self-powered lactate sensor using a ZnO nanowire array modified with LOx. This sensor enables the evaluation of tumor development by detecting lactate concentrations in the tumor microenvironment. The operating principle of this self-powered lactate sensor lies in the synergistic integration of the piezoelectric effect and enzymatic reaction. The sensing process does not require an external power source or batteries. The lactate concentration detection upper limit of the sensor is at least 27 mM, with an LOD of approximately 1.3 mM. The sensor exhibits a response time of approximately 10 s, outperforming most other lactate sensors in terms of speed. Furthermore, the sensor demonstrates superior reversibility, selectivity, accuracy, stability, and repeatability, making it a robust and reliable tool for lactate detection. Preliminary cell experiments confirmed the ability of this lactate sensor to distinguish between cancer cells (4T1-luc) cultured for varying durations (27, 30, 33, 36, 39, 42, 45, and 48 h). In ex vivo experiments with tumor tissues, the device successfully detected tumor tissues of different sizes (100, 150, 300, and 800 mm^3^), indicating that this type of biosensor may assist in further staging cancer and selecting cancer treatment methods.

## Figures and Tables

**Table 1:** Comparison with previously reported methods for lactate detection.

Material	Detection Method	Detection Limit	Response Time	Samples	Extra Power Supply	Ref.
LDH/BSA-capillary	Fluorometric	4.9 μM	10 min	PBS	Needed	[47]
ZnO NPs	Colorimetry	3.98 mM	2–3 min	Tomato sample	Needed	[48]
LDH/SPCE	Impedimetric	0.1 mM	-	Sweat	Needed	[49]
Au/PDA/PtMN	Voltammetry	50 μM	2 min	Serum	Needed	[50]
LOx/Ti_3_C_2_T_x_/MB	Enzymatic catalysis	17.05 μM	20 s	Sweat	Needed	[51]
ZnO/LOx	Piezoelectric–enzyme	1.3 mM	10 s	PBS	Not needed	Present work

LDH, lactate dehydrogenase; BSA, bovine serum albumin; ZnO NPs, zinc oxide nanoparticles; SPCE, screen-printed carbon electrodes; Au/PDA/PtMN, Pt-microneedle that was modified with Au nanoparticle-decorated polydopamine nanospheres; and Ti_3_C_2_T_x_/MB, MXene/methylene blue.

## Data Availability

The experimental data are contained within the article.
